# Insulin eye drops improve corneal wound healing in STZ-induced diabetic mice by regulating corneal inflammation and neuropeptide release

**DOI:** 10.1186/s12886-024-03436-3

**Published:** 2024-04-09

**Authors:** Shudi Chen, Yingsi Li, Wenjing Song, Yu Cheng, Yuan Gao, Luoying Xie, Meiting Huang, Xiaoming Yan

**Affiliations:** Department of Ophthalmology, Peking University First Hospital, No.8 Xishiku Avenue, 100034 Beijing, China

**Keywords:** Insulin eye drops, Diabetic keratopathy, Nerve regeneration, Neuropeptides, Inflammation

## Abstract

**Introduction:**

In recent years, insulin eye drops have attracted increasing attention from researchers and ophthalmologists. The aim of this study was to investigate the efficacy and possible mechanism of action of insulin eye drops in diabetic mice with corneal wounds.

**Methods:**

A type 1 diabetes model was induced, and a corneal epithelial injury model of 2.5 mm was established. We used corneal fluorescein staining, hematoxylin-eosin (H-E) staining and the Cochet-Bonnet esthesiometer to examine the process of wound healing. Subsequently, the expression levels of Ki-67, IL-1β, β3-tubulin and neuropeptides, including substance P (SP) and calcitonin gene-related peptide (CGRP), were examined at 72 h after corneal injury.

**Results:**

Fluorescein staining demonstrated an acceleration of the recovery of corneal epithelial injury in diabetic mice compared with the saline treatment, which was further evidenced by the overexpression of Ki-67. Moreover, 72 h of insulin application attenuated the expression of inflammatory cytokines and neutrophil infiltration. Remarkably, the results demonstrated that topical insulin treatment enhanced the density of corneal epithelial nerves, as well as neuropeptide SP and CGRP release, in the healing cornea via immunofluorescence staining.

**Conclusions:**

Our results indicated that insulin eye drops may accelerate corneal wound healing and decrease inflammatory responses in diabetic mice by promoting nerve regeneration and increasing levels of neuropeptides SP and CGRP.

## Introduction

Neurotrophic keratopathy (NK) is a degenerative disease of cornea with trigeminal nerve damage, characterized by reduced or absent corneal sensation, dry eye, corneal epithelial defects, corneal ulcers, even corneal stromal melt and perforation [1]. Diabetes mellitus (DM) is one of common causes of NK, which imposes a substantial economic burden throughout the world on a yearly basis [2]. The most recent data from the International Diabetes Federation (IDF) estimates that 10.5% of people globally had diabetes mellitus in 2021 [3]. By 2045, this number is expected to increase from 536.6 million to 783.2 million [3]. Long-term environment of higher blood glucose can result in a group of pathologic alterations to the cornea, known as diabetic keratopathy (DK). It has been estimated that more than 70% of DM patients eventually develop DK [4, 5]. There is a possibility that the loss of corneal subepithelial nerves can explain the persisting corneal epithelial abnormalities and decreased rates of repair in diabetic patients. However, at present, such patients who receive no targeted therapy for progressive nerve damage can eventually exhibit suboptimal outcomes.

Peripheral sensory nerves can release neuropeptides, such as substance P (SP), α-melanocyte stimulating hormone (α-MSH), calcitonin gene-related peptide (CGRP) and methionine enkephalin (ME) [6–8]. The cornea has one of the highest concentrations of nerve endings of any organ. These neuropeptides have been found in human tears and animal tears in the past two decades, which is based on research conducted in human beings and in animal models [9–11]. These molecules are released by corneal peripheral sensory nerves and serve as indispensable nutrients for the dynamic balance of the ocular surface environment [6, 9, 12]. In addition to increasing sensory nerve reactivity and stimulating nerve fiber development, neuropeptides can influence the course of the inflammatory response in the cornea [12]. Studies have shown that SP is also a proinflammatory neuropeptide that activates a variety of immune cells, including eosinophils, T cells, dendritic cells, mast cells, macrophages and neutrophils [13]. Moreover, CGRP can block the maturation of dendritic cells, as well as the production of inflammatory components by macrophages [14]. A decreased concentration of neuropeptides has been observed in patient tears with corneal hypesthesia, due to the loss of sensory nerve fiber density [10]. Recent reports have shown that the exogenous neuropeptides SP or vasoactive intestinal peptide (VIP) can increase the epithelial lesion healing rate by regulating nerve regeneration and inflammatory reactions in vivo [15, 16]. As a result, both inflammatory and neurological responses are important to corneal homeostasis regulation during the recovery process of corneal wounds.

In the clinical setting, subcutaneous insulin therapies are of prime importance in managing the fluctuation of blood glucose levels. Unquestionably, one of the most significant scientific breakthroughs that has impacted individual throughout the world over the last century was the discovery of insulin. For the past century, the application of exogenous insulin has been investigated as an effective therapeutic modality for promoting the recovery of cutaneous wounds and burns of diabetic and nondiabetic animals and patients, which has attracted many researchers to this field [17–20]. Its role in wound healing has also been validated in the wounded cornea. In 1945, an ophthalmologist named Aynsley first observed the influence of the application of insulin injections and/or eye drops for treating patients with corneal ulcers [21]. Unexpectedly, the cornea healed at a faster rate than in previous patients. Therefore, increased attention by more physicians and researchers has been given to the effect and mechanisms of topical insulin application. In recent years, several clinical studies have been performed to further explore the efficacy of topical insulin in patients with refractory neurotrophic keratopathy [22, 23], refractory persistent epithelial defects and dry eye in DM [24–26], thus suggesting that insulin eye drops are potential targeted drugs for nerve impairment and corneal epithelial lesions.

To date, few studies have assessed the mechanisms of insulin eye drops in promoting the healing of NK related corneal epithelial wound and nerve injuries [27–29]. In addition, there has been no in vivo study evaluating the interaction of insulin eye drops with neuropeptides and inflammation during corneal healing. Hence, in this study, we established a type 1 diabetes model with peripheral nerve damage induced by streptozotocin (STZ) and scraped the corneal epithelium to establish one NK model to examine how topical insulin can treat corneal epithelial wounds and stimulate nerve regeneration. We also examined how insulin affected the neuropeptides and inflammatory responses of the cornea.

## Materials and methods

### Animals

**Fig. 1:**
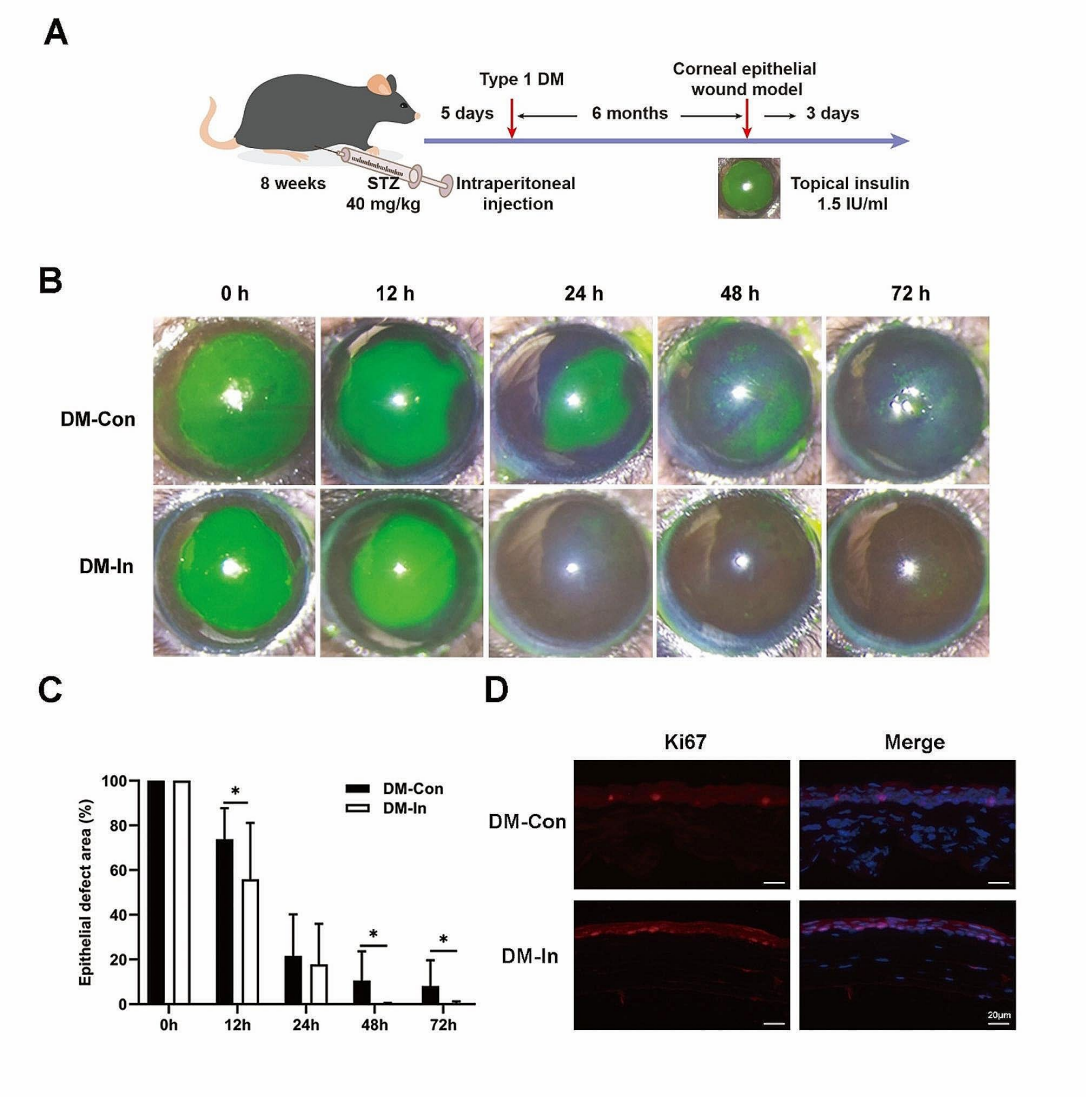
Insulin eye drops promote corneal wound healing and corneal epithelial regeneration. (**A**) Schematic illustration of the experimental protocol. (**B**)Representative images of corneal fluorescein staining of diabetic mice in DM-In group and DM-Con group after the removal of corneal epithelium at 0 h, 12 h, 24 h, 48 h and 72 h. (**C**) The percentage of corneal epithelial defect area at different time points and original wound area in two groups. (*n* = 11 in DM-In group; *n* = 14 in DM-Con group). Data are expressed as mean ± SD. (**D**) Representative immunofluorescence images of Ki67 (red, white arrows) costaining with DAPI (blue) in diabetic cornea between two groups. Scale bars: 20 μm. STZ: streptozotocin; DM: Diabetes mellitus; DM-In, diabetic mice with topical insulin treatment; DM-Con, diabetic mice with topical physiological saline treatment; Ep, corneal epithelium; St, corneal stroma

For this experiment, we used C57BL/6 male mice at 8 weeks of age, which were obtained from SPF Biotechnology Co., Ltd., (Beijing, China), and housed them in a barrier environment in the laboratory. In addition, the Laboratory Animal Ethics Committee approved the experimental procedures in accordance with the ARVO Statement for the Use of Animals in Ophthalmic and Vision Research. During the experiment, STZ (40 mg/kg) was intraperitoneally administered for 5 consecutive days to adult C57BL/6 mice to induce type 1 DM, which was consistent with the procedure of a previous study [15]. The OneTouch blood glucometer was used to measure blood glucose levels in the caudal tip vein of mice. In our study, mice with blood glucose levels ≥ 12.0 mmol/L were considered to have type 1 DM and were used in the subsequent experiments. Subsequently, these diabetic mice were fed a standard-fat diet for 6 months. At the conclusion of the experiment, mice were euthanized by cervical dislocation. The specific experimental design was shown in Fig. 1A.

### Corneal epithelial wound and treatment

After six months of standard-fat feeding, we used type I diabetic mice that were successfully induced by STZ for corneal epithelial wound model establishment. After injecting 0.5% sodium pentobarbital solution into the abdomen, 0.4% oxybuprocaine hydrochloride was topically applied under general anesthesia. The operation was subsequently performed by using a corneal ring drill (2.5 mm in diameter), which was first performed to gently press the central area of the cornea to form an indentation. Subsequently, the central corneal epithelium was scraped off with a corneal remover (Algerbrush II, USA) without corneal stroma injury according to the location of the indentation. Afterwards, all of the mice with successful operations were randomly divided into two groups: diabetic group + topical physiological saline (DM-Con) and diabetic group + topical insulin eye drops (DM-In). For topical insulin application, the regular Lysergic insulin injection (Unisom, Lilly France) and saline were combined to create an insulin drop with a concentration of 1.5 IU/mL, and was applied for 72 h at 4 times a day; the same amount of 0.9% sodium chloride solution was used in the control group. The insulin concentration was determined through our preliminary experiment.

### Corneal sensitivity measurement

An esthesiometer Cochet-Bonnet (Luneau Ophtalmologie, France) was used to measure the changes of corneal sensitivity in the central cornea, in accordance with earlier studies [15, 30, 31]. Broadly, the nylon filament was gradually reduced by 0.5 cm at each time from its maximum length (60 mm) in the esthesiometer until a favorable blink reflex was triggered. Each mouse was tested by a researcher at 0 h and 72 h after the topical treatment in each group. Furthermore, the value of corneal sensitivity was verified three times, and the average value of the longest fiber lengths was considered the final result.

### Corneal fluorescein sodium staining

After the operations of the mice, we recorded the wound repair progress on the wounded corneas of the diabetic mice by dribbling 1% sodium fluorescein solution into the conjunctival sac. The photographs were assessed under cobalt blue light at 0 h, 12 h, 24 h, 48 h and 72 h after the surgery. Subsequently, ImageJ software (version 1.8.0, USA) was used to quantify defects in the corneal epithelium at each time point. Finally, the percentages of corneal epithelial lesion area at different time points (12 h, 24 h, 48 h and 72 h) and 0 h were determined.

### Hematoxylin-eosin (H-E) staining

Briefly, all of the mice were euthanized at 72 h after the treatment, and whole eyeballs (*n* = 3) were quickly harvested and embedded in Tissue-Tek O.C.T (Sakura Finetek, Tokyo, Japan). The corneal tissues were then cut into 7 μm thick frozen sections. Each section had three tissues, and the tissues were then stained with hematoxylin and eosin (Seville, China).

### Corneal whole-mount staining immunofluorescence staining

After treatment for 72 h, the mice were sacrificed, and the eyeballs (*n* = 3) were enucleated and subsequently fixed in 4% immunohistochemical fixation solution for 2 h. After rinsing with phosphate buffer solution (PBS) three times, all of the corneas were clipped along the margin of the keratosclera and then incubated with anti-β3-tubulin (1:500, ab52623, Abcam, Cambridge, United Kingdom), anti-SP (1:400, ab10353, Abcam) and anti-CGRP (1:400, 14959s, Cell Signaling Technology, United Kingdom) for 48 h at room temperature. After washing with PBS, the cornea was incubated with Alexa Fluor® 488 goat anti-guinea pig IgG H&L (1:500, ab10353, Abcam) and Alexa Fluor® 594 goat anti-rabbit IgG H&L (1:400, ZF-0516, ZSGB-BIO, China) at 4 °C overnight. After counterstaining with 4,6-diamidino-2-phenylindole (DAPI, ZSGB-BIO, China) to stain the nuclei, all of the images were captured by using a fluorescence microscope (Nikon, Tokyo, Japan). To compare the changes in corneal innervation, Image J and Neuron J software were used to calculate the nerve length in the positive area for β3-tubulin staining per sample. In the experiment, one representative image in the center of the cornea and four replicates captured in the peripheral area of cornea from three independent experiments per group were used for analysis.

### Immunofluorescence staining

The tissues were taken 72 h after the injury to the cornea. First, we rewarmed the sagittal frozen sections of corneas at room temperature for 5 min and fixed them with 4% immunohistochemical fixative for 20 min after removing them from the 80 °C temperature. Subsequently, 0.1% Triton solution was added for permeation for 20 min, and 5% BSA (Beyotime, China) was added for 1 h. The cells were incubated with anti-IL-1β (1:200, Abcam), anti-Ki67 (1:200, ab16667, Abcam), anti-SP (1:200, ab10353, Abcam) and anti-CGRP (1:400, 14959s, Cell Signaling Technology) antibodies at 4 °C overnight. Finally, all of the sections were incubated with diluted fluorescent secondary antibody for 1 h. Then, all of the images were captured and analyzed.

### Statistical analysis

The software SPSS version 24.0 (IBM Corporation, Chicago, IL) was used for the data analysis. All of the data (corneal epithelial injury area, corneal sensitivity, corneal nerve density and corneal neuropeptide density) are expressed as the mean ± standard deviation ($\stackrel{-}{x}\pm s$). The data between the two groups were analyzed by using the nonpaired Student’s t test, and the value of *P*<0.05 was considered to be statistically significant.

## Results

### Insulin eye drops promote corneal wound healing and corneal epithelial regeneration

As Fig. 1A shown, diabetic mice were given 1.5 IU/mL insulin eye solutions at 4 times daily for 3 days to determine how the drops affected the healing of corneal wounds. According to the corneal fluorescein staining, the results showed that corneal epithelial injury was almost healed at 48 h in the DM-In group, whereas intense punctate staining was observed in the DM-Con group at 48 h and 72 h (shown in Fig. 1B). As shown in Fig. 1C, there were significant differences in corneal epithelial defects between the DM-In group and the DM-Con group at 12 h, 48 h and 72 h (*p* < 0.05). Ki67 is a common marker of corneal epithelial proliferation. Consistent with previous studies [18, 27], the application of insulin eye drops contributed to an increased level of Ki67 at 72 h in the corneal epithelium compared to the DM-Con group (shown in Fig. 1D). Taken together, these results suggested that insulin eye drops can accelerate the healing of corneal wounds in mice with DK and more rapidly regenerate the epithelium.

### Insulin eye drops attenuate inflammatory responses in corneal epithelium

**Fig. 2:**
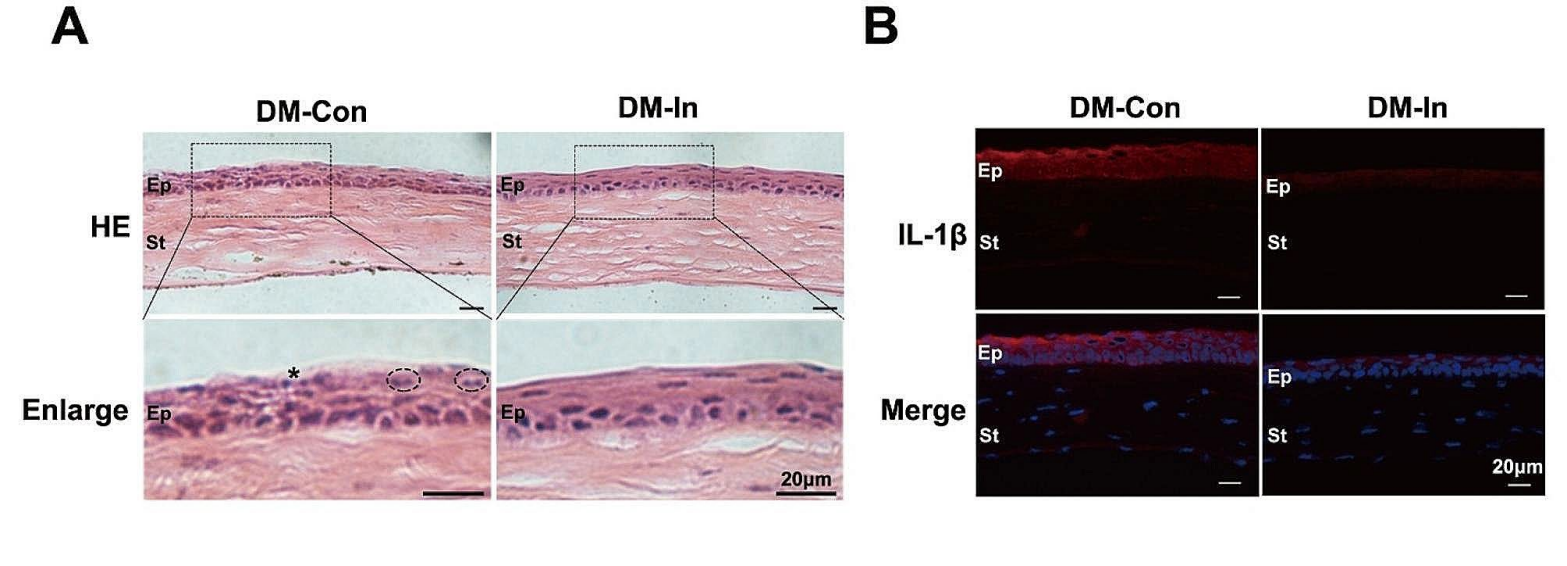
Insulin eye drops attenuate inflammatory responses. (**A**) The typical histopathological changes of diabetic cornea by HE staining in DM-In group mice and DM-Con mice at 72 h (*n* = 3). Asterisk mark: lymphocytes; black dotted circle: segmented granulocytes. Scale bars: 20 μm. (**B**) Representative Il-1β (red) expression in corneal epithelium was examined by immunofluorescent techniques at 72 h (*n* = 3). In the sagittal frozen sections of corneas, Il-1β expression was found mostly within corneal epithelium. Scale bars: 20 μm. DM-In, diabetic mice with topical insulin treatment; DM-Con, diabetic mice with topical physiological saline treatment; Ep, corneal epithelium; St, corneal stroma

As part of our investigation into the effect of insulin on inflammatory responses in diabetic mice, we collected frozen sections of the cornea and stained them with H-E. A decreased number of inflammatory cells (such as lymphocytes and leukocytes) were observed in the diabetic corneal epithelium after treatment with insulin compared with the DM-Con group (shown in Fig. 2A). Consistently, representative immunofluorescence staining showed a remarkable reduction in the expression of the typical proinflammatory factor IL-1β in the DM-In group compared with the DM-Con group (shown in Fig. 2B).

### Insulin eye drops improve corneal nerve regrowth in diabetic mice with corneal lesions

**Fig. 3:**
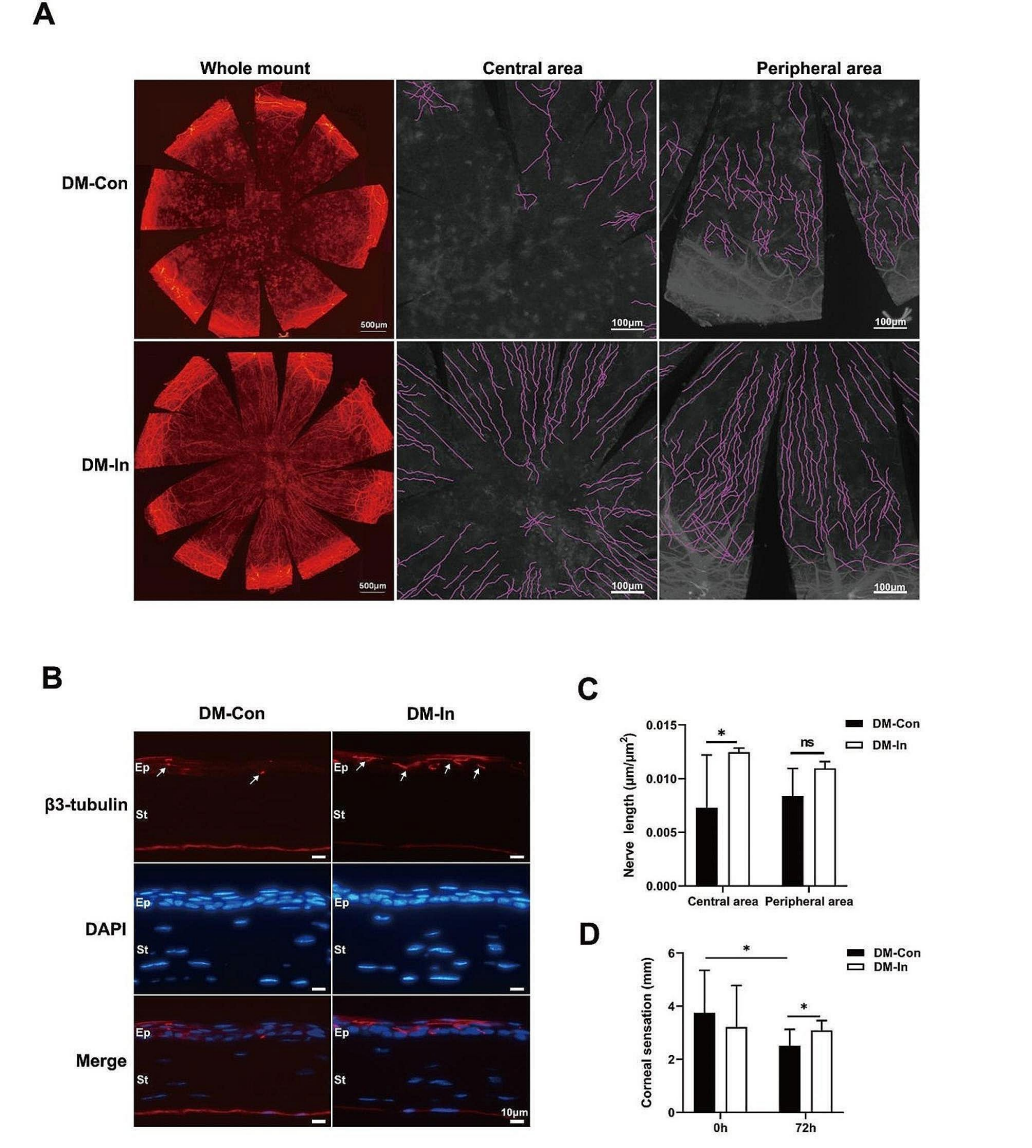
Insulin eye drops improve corneal nerve regeneration. (**A**) The typical changes of regenerated nerve fibers in the cornea after the administration of insulin by using β3-tubulin (red), a pan-neuronal marker, immunofluorescence in the whole-mount cornea (*n* = 3). (**B**) Representative corneal cross-sections further show the locations and the regeneration (white arrows) of the central corneal epithelial nerve costaining with β3-tubulin (red) and DAPI (blue), (*n* = 3). (**C**) The length of corneal nerve fibers (µm/µm2) in the central and peripheral areas was calculated using Neuron J software. (**D**) The dynamic changes of corneal sensation (mm) in two groups at 0 h and 72 h. Data were exhibited as mean ± SD; *P < 0.05. Scale bars: 500 μm, 100 μm, and10µm. DM-In, diabetic mice with topical insulin treatment; DM-Con, diabetic mice with topical physiological saline treatment; Ep, corneal epithelium; St, corneal stroma

To examine how nerve regrowth following corneal epithelium loss was affected by insulin eye drops, we examined the presented alterations in the corneal nerve stained with the neuronal marker β3-tubulin. As shown in Fig. 3A shown, although the normal swirling structure of nerve fibers in the central area were not observed in the DM-in group, the central corneal nerve fibers recovered quickly, faster than those in the DM-Con group. Figure 3B showed representative cross-section images in the central corneal in different groups. Using Neuron J software, the regeneration of corneal nerve fibers in the central and peripheral areas were detected. As shown in Fig. 3C, the nerve length in central cornea was significantly increased in the DM-In group compared with the DM-Con group (12.47 ± 0.39 vs. 7.29 ± 4.90 10^− 3^ μm/µm^2^, *P* < 0.05). However, there was no significant difference in the length of peripheral nerve fibers between groups. Moreover, a considerable decline in corneal sensitivity was observed after epithelial removal (3.75 ± 1.53 vs. 2.5 ± 0.58 mm, *P* < 0.05), whereas topical insulin therapy was observed to keep corneal sensitivity from decreasing (shown in Fig. 3D). Collectively, these results suggested that insulin eye drops can restore corneal nerve regeneration that was impaired after the damage to the corneal epithelium.

### Insulin eye drops increase levels of the neurotrophic factors in the cornea of diabetic mice

**Fig. 4:**
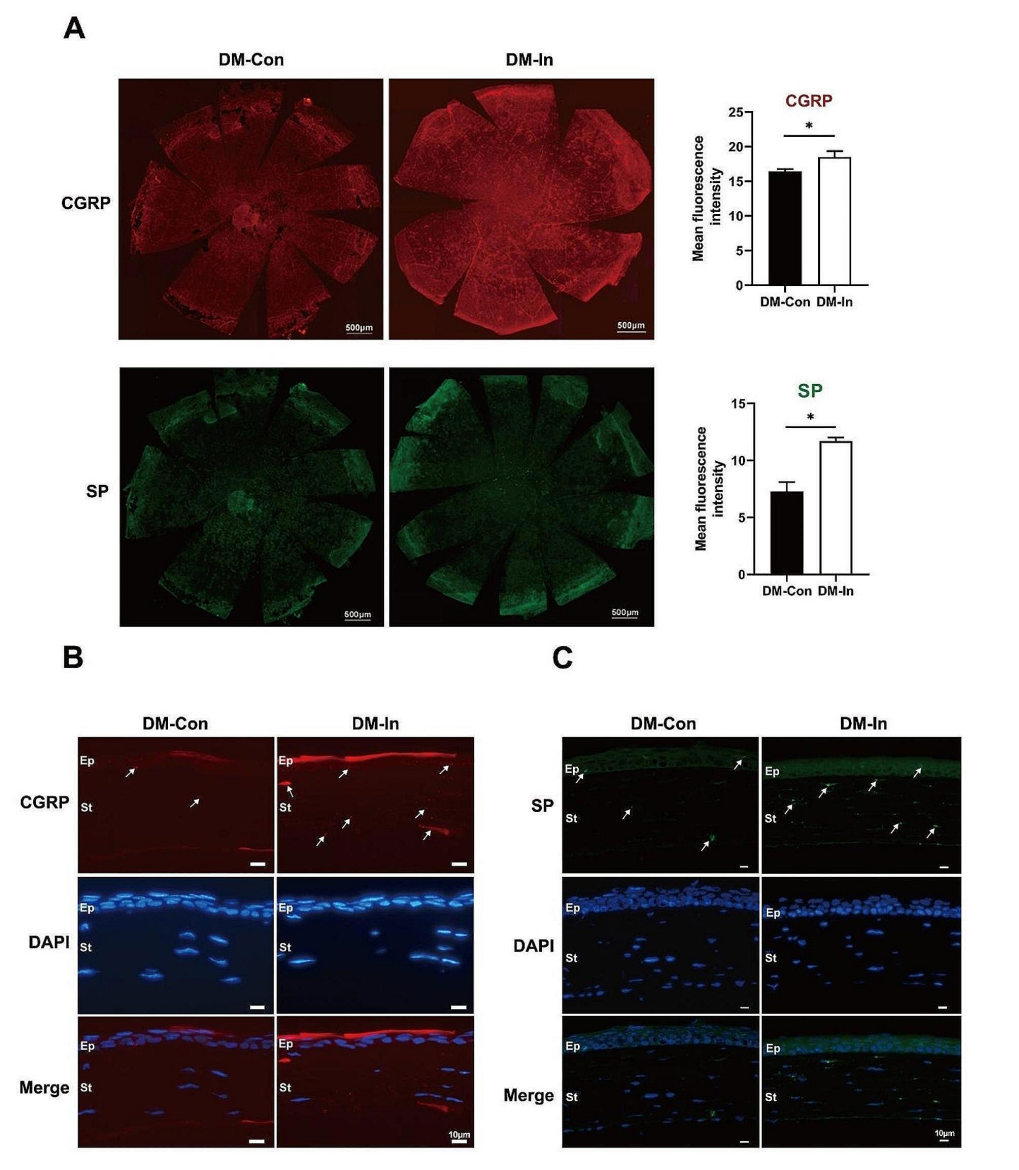
Insulin eye drops enhance expression levels of the neuropeptides. (**A**) Representative images of immunofluorescence staining of the whole cornea labeled with CGRP (red), SP (green) and DAPI (blue) (*n* = 3). The intensity of neurotrophic factors in the whole-mount cornea were quantified by Image J software. Data were exhibited as average ± SD; *P < 0.05. (**B**) and (**C**) are representative images of the corneal sections, showing the positions of CGRP (red) and SP (green), as indicated by the white arrows. Scale bars: 500 μm, 100 μm, and10µm. DM-In, diabetic mice with topical insulin treatment; DM-Con, diabetic mice with topical physiological saline treatment; Ep, corneal epithelium; St, corneal stroma

Recently published studies have shown that topical neurotrophic factors could promote the regeneration of corneal fibers and the repair of corneal epithelial [16, 32]. In the experiment, we detected the expressions of neurotrophic factors including SP and CGRP in the cornea via corneal immunofluorescence staining. Of note, enhanced expression levels of the neuropeptides SP (11.7 ± 0.26 vs. 7.26 ± 0.69, *P* < 0.01) and CGRP (18.51 ± 0.69 vs. 16.45 ± 0.25, *P* < 0.05) in the cornea were observed in DM-In group mice compared with DM-Con group after 3 days of topical insulin treatment (shown in Fig. 4A). Sagittal frozen sections of corneas stained with CGRP or SP showed that neuropeptides were mainly expressed in corneal epithelium and stroma (shown in Fig. 4B-C). Our results suggested that insulin eye drops restore corneal nerve regeneration with concomitant increase in neuropeptide expression.

## Discussion

In our study, insulin eye drops and saline treatment for corneal injury healing were investigated by using type 1 DM mice to establish one model of NK. Specifically, we focused on corneal sensitivity, histological changes, inflammatory processes, corneal nerves and neuropeptide expression in wounded corneas after topical insulin treatment for 72 h. DM-related corneal damage is often accompanied by corneal epithelium irregularity and damaged corneal sensation. According to our findings, 1.5 U of topical insulin promoted corneal epithelial and corneal nerve regeneration in wounded areas of DM mice, as has been previously reported [24, 25, 27]. According to earlier research, FGLM-amide and IGF-1’s C-domain peptide may work in concert to accelerate corneal wound healing. The proinsulin C peptide, however, did not have the same effect [33]. It has been discovered in earlier research that insulin, perhaps through binding to receptors and initiating the PI3K/AKT signaling pathway, can inhibit apoptosis and inflammation while promoting corneal epithelial cell proliferation and migration [34]. Also, studies have shown that by keeping the mitochondrial membrane polarized, insulin encourages the metabolic regulation of corneal epithelial cells, which may then encourage the migration of corneal epithelial cells [35]. As a result, we speculated that the underlying mechanism could help diabetic corneal lesions to heal more rapidly by relieving inflammation and enhancing neurotrophic factors.

Inflammatory cytokines have a dual role in the corneal epithelial wound healing process. During the initial phases of inflammation, corneal Toll-like receptors are triggered, leading to the release of pro-inflammatory mediators and the recruitment of neutrophils [36]. These processes eliminate pathogenic microorganisms and facilitate the healing of corneal epithelial wounds. For instance, the inflammatory cytokine IL-6 has been shown to support corneal epithelial wound healing, as reported by Nishida T et al. [37] However, dysfunctional resolution of the undue inflammatory response also could result in reduced lesion healing in the cornea. Cytokines of the IL-1 class (such as IL-1α and IL-1β) mediate inflammatory and innate immune responses [38]. On the one hand, IL-1 could stop germs from spreading by causing the corneal cells that are already infected to undergo apoptosis [39]. Conversely, IL-1, a proinflammatory factor, can also stimulate antigen-presenting cells, inflammatory cells, and chronic inflammation in the cornea. Previous studies have confirmed that increased IL-1β in diabetic corneas leads to the overexpression of matrix metalloproteinases, apoptosis of stromal keratocytes and delayed corneal epithelial wound healing [38, 40]. Our findings demonstrated that the topical application of 1.5 U insulin inhibited the expression of proinflammatory IL-1β in diabetic corneas in comparison with mice without insulin treatment. Coincidently, we detected a decreased number of inflammatory cells (such as lymphocytes and leukocytes) infiltrating the diabetic corneal epithelium after treatment with insulin. Therefore, we speculate that IL-1 may play a secondary regulatory role in this process. Similarly, a previous study confirmed that local treatment with insulin cream considerably improved thermal burning wound healing in diabetic rats by modulating the recruitment of inflammatory cells, such as monocytes/macrophages, and the expression of proinflammatory cytokines [18]. Thus, these findings suggested that one of the underlying mechanisms by which topical insulin accelerates corneal wound healing is related to inflammation reduction.

SP and CGRP are widely reported neuropeptides that are present in the nervous system [6]. They have also been reported to exhibit a protective role in diabetic corneas by regulating the inflammatory response on the ocular surface and by clearing corneal ROS accumulation and promoting mitochondrial function recovery [6, 15, 16, 41]. However, in patients with NK and corneal ulcers, the loss of corneal sensory nerves results in a decreased concentration of SP in tears [10]. Our study revealed that insulin eye drops augmented the expression of the neuropeptides SP and CGRP in the healing corneas. Our hypothesis was that the discharges of SP and CGRP in turn nurtured the corneal nerve, thereby increasing corneal sensitivity and promoting corneal nerve regeneration as well as corneal epithelial wound closure. In addition, the ability of CGRP and SP to effectively regulate the inflammatory response in local tissues has been well established in previous studies [13, 14]. Therefore, we hypothesized that the anti-inflammatory effect of topical insulin may be achieved by controlling the production of the neuropeptides CGRP and SP. However, the lack of clarity regarding the precise role that insulin played in these significant and intricate interactions between immune, neural, and epithelial cells was one limitation of this experiment. Secondly, whether the increase in CGRP and SP was a consequence of nerve regeneration or one of the mechanisms of insulin action was unknown. The tolerance and safety of 1.5 U insulin for mice should also be further observed.

In conclusion, this study evaluated the effect of topical insulin application on the ocular surface by using a mouse model of DK, and our results indicated that insulin eye drops may accelerate corneal wound healing and decrease inflammatory responses in diabetic mice by promoting nerve regeneration and increasing levels of neuropeptides SP and CGRP.

## Data Availability

All data generated or analyzed during this study are included in this article. Further enquiries can be directed to the corresponding author.
